# Supplementary figures and images for: Impact of Nitrogen Fertilizer Levels on Metabolite Profiling of the Lycium barbarum L. Fruit
Source: Molecules. 2019 Oct 28;24(21):3879. doi: 10.3390/molecules24213879 (PMC6864581; doi:10.3390/molecules24213879)

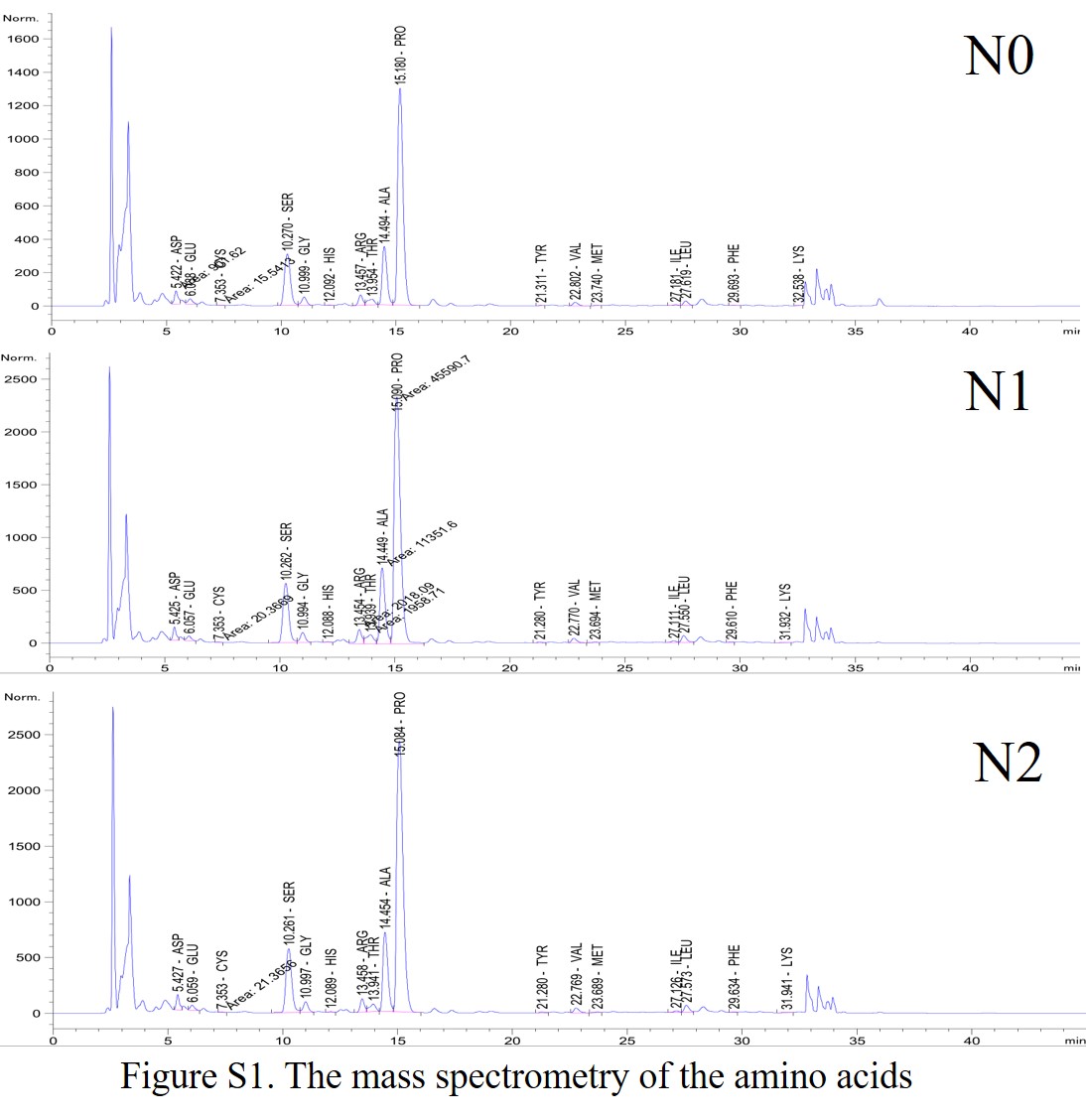

Supplement: Supplementary file 1 [file molecules-24-03879-s001.zip › FIgure S1.jpg]

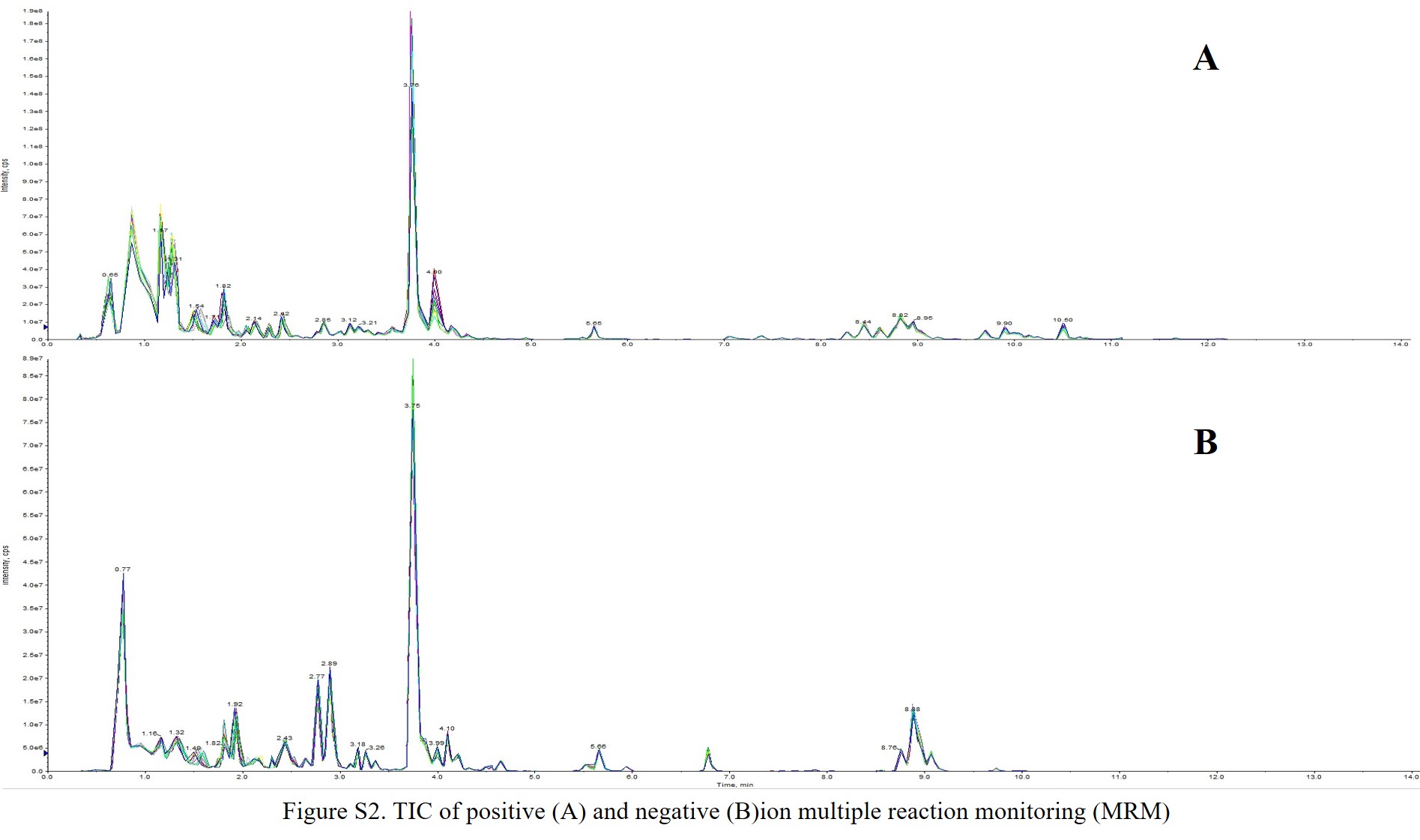

Supplement: Supplementary file 1 [file molecules-24-03879-s001.zip › FIgure S2.jpg]
